# Using online adverts to increase the uptake of cervical screening amongst “real Eastenders”: an opportunistic controlled trial

**DOI:** 10.1186/1756-0500-6-117

**Published:** 2013-03-26

**Authors:** Ray B Jones, Mar Soler-Lopez, Daniel Zahra, Judith Shankleman, Esther Trenchard-Mabere

**Affiliations:** 1 Faculty of Health, Plymouth University, 3 Portland Villas, Plymouth PL4 8AA, UK; 2 NHS East London and the City, Tower, Hamlets Public Health, 4th Floor, Aneurin Bevan House, 81, Commercial Road, London, E1 1RD, UK; 3 Department of Psychology, Plymouth University, Portland Square, Plymouth, Pl4 8AA, UK

**Keywords:** Cervical screening, Online advertising, Media, Television drama

## Abstract

**Background:**

Cervical screening uptake has increased as a result of occurrences of cervical cancer in TV ‘soap operas’ and in real life celebrities such as Jade Goody. Media analysis at the time of Jade Goody’s death suggested the NHS did not take sufficient advantage of this opportunity to improve cervical screening rates. Google AdWords has been used to recruit and raise awareness of health but we were not aware of its use to supplement media events.

**Methods:**

This was an opportunistic service evaluation to accompany a cervical cancer storyline in Eastenders (a TV ‘soap opera’). We ran an AdWords campaign based on keywords such as ‘Eastenders’, and ‘cervical cancer’ in a one mile radius in East London, linked to one webpage giving details of 10 practices and other links on cervical cancer. We recorded costs of adverts and setting up the webpage. We used routine statistics from Tower Hamlets, City and Hackney, and Newham Primary Care Trusts (PCTs) of the number of smears, eligible populations, and coverage by practice by month from September 2010 to January 2012 to compare the ten intervention practices with controls.

**Results:**

Eight people per day in the target area viewed the project webpage. The cost of setting up the website and running Google AdWords was £1320 or £1.88 per person viewing the webpage. Unlike Jade Goody’s death, there was no major impact from the Eastenders’ storyline on Google searches for cervical cancer. There was considerable monthly variation in the number of smear tests in the 3 PCTs. The AdWords campaign may have had some effect on smear rates but this showed, at best, a marginal statistical difference. Assuming a ‘real’ effect, the intervention may have resulted in 110 ‘extra’ women being screened but there was no change in coverage.

**Conclusions:**

Although the Eastenders storyline seemed to have no effect on interest in cervical cancer or screening, the AdWords campaign may have had some effect. Given the small scale exploratory nature of the study this was not statistically significant but the relatively modest cost of advertising suggests a larger study may be worthwhile. An outline of a possible study is described.

## Background

Cervical cancer is mostly caused by persistent infection with high-risk human papilloma virus which causes changes in the cells covering the cervix that make them more likely to become cancerous in time. Although due to the national cervical screening programme in the United Kingdom (UK), cervical cancer is now relatively rare representing just 2% of all cancers in women, it is the most common cancer in females under 35. Over the period 2007–2009 a yearly average of 3100 women were diagnosed with cervical cancer and nearly 1,000 women died from the disease [1]. Pre-cancerous cells can take many years to develop into cancer, and early detection through cervical screening is important in preventing the development of these pre-cancerous cells into cancer and also in the success of treatment once cancer has already developed [2]. It was estimated in 2004 that the cost per life saved was £36000 [3].

In the English National Health Service (NHS), cervical screening is offered to all women aged 25–64, every three or five years depending on their age. Audits of cervical cancer show that the biggest risk factor for developing cervical cancer is non-attendance to screening; many of those who develop cancer have never been screened [2]. The effectiveness of the programme is assessed among other parameters, by its coverage rate, the percentage of women aged 25–64 who have been adequately screened within a period of five years. The target for overall coverage is 80% [4].

Cervical screening uptake has been shown to increase as a result of occurrences of cervical cancer in TV ‘soap operas’ [5,6] and in real life celebrities such as Jade Goody [7-9]. However, analysis of the media at the time of Jade Goody’s death [8,9] also suggested that the NHS did not take sufficient advantage of this opportunity to try to improve cervical screening rates and health promotion. We learned in the summer of 2011 that a character (Tanya Branning) in the TV soap opera ‘Eastenders’ was about to be diagnosed with cervical cancer. Indications from a Google search on ‘Eastenders’, ‘Tanya Branning’ and ‘Cervical Cancer’ were that the NHS (e.g. NHS North East Essex on Facebook [10]) and relevant charities (e.g. Jo’s Trust [11]) were preparing to capitalise on the media interest by providing links on websites to cervical screening information.

Google AdWords (AdWords) has been used by others to recruit to studies [12-17], and we had used it to raise awareness of online therapies for depression [18] with location targeted adverts at postcode area level. Our experience was that this location targeting ‘leaked’ to neighbouring areas to some degree [18] but it seemed worth exploring further. We were not aware of the use of paid-for online advertising to use media events for health promotion.

This was an opportunistic exploratory controlled trial to see if online advertising to encourage uptake of cervical screening at the time of a TV fictional ‘event’ would be read and acted upon, and if it was feasible to assess impact on ten practices in one location compared to other practices in three East London Primary Care Trusts (PCTs).

## Methods

### Service evaluation

This project was an opportunistic service evaluation; we did not recruit participants, but used routine data to assess impact. Ethics permission was therefore not sought. Google Analytics (Analytics) data and the log file from the project website, both set up especially for this project, are available from the authors. Uptake and coverage of cervical screening by general practice is publicly available. It is not (yet) routinely published online by the NHS. Anonymous data is available from the authors on request. It is likely that in due course this type of data will be published online (http://www.guardian.co.uk/politics/2012/jun/24/cancer-patients-online-data-gps).

### Intervention

**Figure 1:**
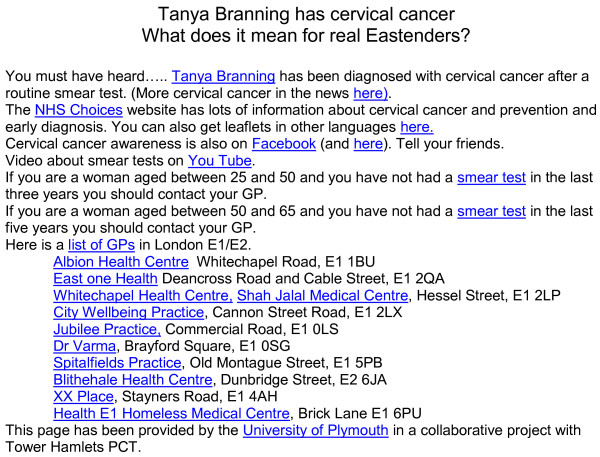
One page website to which participants were directed from Google advert.

We set up a location limited (one mile radius around E1 1BU) AdWords campaign based on keywords such as ‘Eastenders’, and ‘cervical cancer’. Google decided when to present adverts based on the estimated location of the user and a match between the search terms entered, the keywords specified, and competing bids for adverts. Those who clicked on the advert were directed to a one page website (Figure 1). The webpage re-iterated information about the location and had links to further information about cervical cancer and screening, and links to 10 practices in that area (Additional file 1). This postcode was originally chosen to try to obtain the maximum distance between an intervention and control area within Tower Hamlets PCT. Subsequently we were able to add data from two neighbouring PCTs to the study enabling a larger control area.

**Figure 2:**
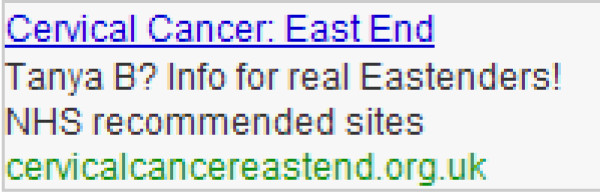
Original Google advert run from 24th July to August 3rd 2011.

Originally a single advert (Figure 2) was run from 24th July to August 3rd 2011 but, on the advice of Google support, the campaign was changed. From 3rd August 13 variations of advert were tried (Additional file 1). The budget was originally set at £10/day, raised to £12/day on 10th August and to £15 on 16th August.

### Outcomes

We used data from AdWords, Analytics for our website, and our website captured date, time, and choices made by anonymous users. We used routine statistics from Tower Hamlets, City and Hackney, and Newham PCTs of the number of smears, eligible populations, and coverage (percentage of women screened in the last 5 years (age 50–64) or last 3 years (age 25–49)), by practice and by month from September 2010 to January 2012.

### Study population

Tower Hamlets PCT had 36 practices, City and Hackney 46, and Newham 64 involved in cervical screening. During the period of study the mean total population of women eligible for cervical smears was 232,412 comprising 71789 from Tower Hamlets, 76786 from City and Hackney, and 83837 from Newham. All Newham practices were in control area, City and Hackney was divided between buffer and control and the ten intervention practices were in the western part of Tower Hamlets (Additional file 1).

Cervical screening coverage rates in these three PCTs are below the national average of 80%. The three PCTs have population characteristics that have been shown to be consistent predictors for lower uptake of screening, namely, ethnically diverse, high levels of social deprivation, and high mobility (Additional file 1). The PCTs’ cervical screening teams are therefore, continually striving to increase uptake by implementing a multi-faceted approach of diverse evidence based interventions. In particular, the “12 weeks action plan” intervention in six practices (three of which were in the target group in this study) in Tower Hamlets, between October 2010 and March 2011, showed some short term success in improving coverage rates (internal report MSL, JS, ETM).

### Analysis

Distances of practices from E1 1BU were found from Freemap Tools [19]. We know from other work [18] that location targeted AdWords ‘leak’ in that targeting is not particularly ‘specific’. Leakage may vary between urban and rural areas and between London and other urban areas as estimates of the location of Internet users vary. Within the limitations of our data set we have explored this ‘categorically’ by defining three groups of practices: (1) 10 target practices listed on the website (target zone), (2) practices in a ‘buffer zone’ (we repeated the analysis with buffer zones of 2.5 miles or less and 3.5 miles or less, from the target postcode) but not listed on the website, (3) practices outside of the buffer zone designated as ‘controls’. We also examined the impact using distance from the epicentre of the advert as a continuous variable.

**Figure 3:**
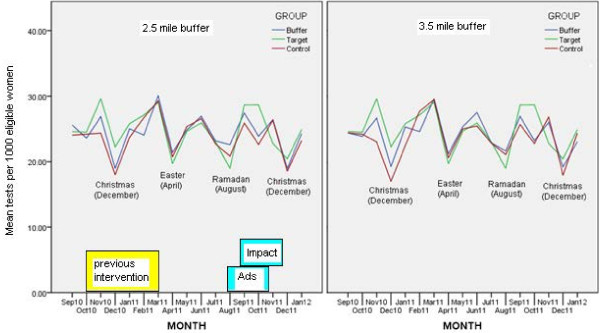
Mean number of smear tests per month carried out per 1000 eligible women for target, buffer, and control groups (left hand graph, buffer set at 2.5 miles, right hand graph buffer set at 3.5 mile).

We plotted (Figure 3) the smear rates per month per 1000 eligible women over a 17 month period to show the annual variation in screening affected by holiday periods. To prevent confounding with the previous “12 weeks action plan” intervention in Tower Hamlets (October 2010 – April 2011) we have further analysed data only from May 2011 to January 2012. In analysis of variance we excluded a ‘buffer zone’, that is, practices near the ‘epicentre’ of the geo-located advert, and compared the 10 target practices with the remaining practices in these three PCTs beyond the buffer zone. We repeated this for buffer zones 2.5 and 3.5 miles from the epicentre (Additional file 1).

First, we compared mean smear rates over the nine months, between target and control including practice as a covariate. If the intervention had an impact we would expect there to be a significant interaction with month such that the difference between target and control groups was increased in September, October, and November 2011 (supposed intervention impact months). Lastly, we carried out three separate analyses of variance to compare screening rates between the target and control groups for September-November (the months with the assumed impact).

To assess overall online interest in cervical cancer we examined Google searches relating to cervical cancer using Google Insights for Search, a database of all Google searches which can be analysed by week of access and country of user [20]. Insights does not provide absolute numbers of searches but a relative figure based on search activity for the time period under study. The week in the selected period is assigned the value 100, and other weeks are scaled accordingly. This was the main data source for a previous study of the ‘Jade Goody effect’ [9]. We examined searches for ‘cervical cancer’ in the health category for the UK.

## Results

### AdWords and Analytics reporting of clicks on advert

In total there were 798 clicks on the adverts between 24th July and 22nd October, from 22334 impressions (presentations) of the adverts, a click through rate of 3.6%. Users had entered a total of 358 different search terms to trigger the presentation of the advert. Search terms were divided into three groups (Table 1). Most (87%) searches were related to cervical cancer with no mention of Eastenders. Analytics recorded 821 visits to the website, 808 from the UK and 13 from overseas (probably web ‘crawlers’ that index websites for search engines). (Analytics naming of locations, particularly in London, is rather idiosyncratic; see [18] for more discussion). The 808 from UK were 586 from ‘Poplar’ (the nearest Analytics location to our target), 109 from ‘London’, 17 from Lambeth, 5 from Kensington and 91 from elsewhere in the UK. In summary, we might estimate that in 12 weeks about 700 people (those located as London) were in the target area (about 8 people per day) and perhaps another 100 outside the area, clicked on the website. One in ten (84) visits were by people using mobile devices.

**Table 1:** Types of user-entered searches, showing clicks on adverts for each of three groups and examples of user entered searches in each group

**Group**	**Clicks**	**Examples of search terms entered**
Eastenders and TV related no mention of cancer	75 (9.4%)	Eastenders, Tanya Branning, Tanya Eastenders
Cancer/health related no mention of Eastenders	690 (86.5%)	Cervical cancer, smear test, cervical cancer symptoms, what is cervical cancer, signs of cervix cancer, how do you detect cervical cancer
Related to Tanya Branning and her health/cancer	33 (4.1%)	Eastenders Tanya cancer, does Tanya die in eastenders, what’s wrong with Tanya in eastenders, what cancer does Tanya have
**ALL**	**798**	

Our website log showed that 116 (14% of 808) clicked through to topics listed on the website (excluding ‘topics’ of Plymouth University and Towers Hamlets PCT). Most (45) of those ‘clicking through’ clicked through to details from NHS Choices (Figure 1), 20 were interested in hearing more about Tanya Branning, 25 more about cervical cancer in the news, eight to cervical awareness on Facebook, seven to a video about smears on YouTube, four on smear test, and seven to one of the GP links.

### Cost modelling

The charge for setting up the project website and domain name was £150. In total the AdWords campaign cost £571.18, comprising £82.76 for the first period (24th July to August 3rd) and £488.42 from August 3rd to the end on 22nd October. The campaign ran for 91 days at an average cost of £6.28 per day. If this was completed as service provision we might estimate 40 hours of research support time (at £15/hour) is needed in liaising with website developers, arranging AdWords and Analytics. The total cost of approximately £1320 for 700 in the target area represents £1.89 per person viewing the one page advert, and £11.38 per person that clicked through to topics from the website. The daily AdWords spend ranged from zero to £12.94, depending on the number of people clicking on the advert each day, with an average daily cost of £6.28. Website and development costs would not increase with a longer campaign, only AdWords costs. As a short exploratory study, to keep costs to a minimum we ended the AdWords campaign after 3 months before the Eastenders cervical cancer story had completed. If we assume that a campaign lasting 6 months had the same number of daily visitors to the website the cost per visitor reduces to £1.35 per person viewing the advert and £8.30 per person clicking through to a topic from the website.

### Scale of impact

To see if the scale of intervention (i.e. this number of people clicking on adverts) could have an impact we compared it with the number of smears taken. The mean number of smear tests for the ten target practices was 847/month between September 2010 and September 2011, so two measures of possible impact of the AdWords campaign are the number seeing the advert as a percentage of the total number of smears during those three months (700/(3*847) = 28%), and the number seeing the advert and clicking on a link as a percentage (116/(3*847) = 5%).

### National impact of the Eastenders’ storyline

**Figure 4:**
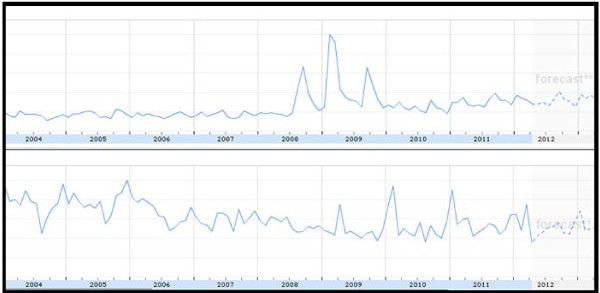
Frequency of Google searches on ‘Cervical Cancer’ (top) and on ‘Eastenders’ for the period 2004–2012 (bottom).

Figure 4 shows that the interest level in cervical cancer in the UK over the last few years peaked during the Jade Goody years (2008–2009). There was no major impact from the Eastenders’ storyline in the latter half of 2011. Interest in Eastenders was average during the study period.

### Comparison of national interest in cervical cancer and clicks on advert

Comparison of Insight with AdWords data showed that clicks on our advert in the one mile target area largely corresponded with national interest in cervical cancer.

### Impact on number of cervical smears taken

Figure 3 shows the considerable variation in the number of smear tests over one year, in particular the number of smears taken at Easter, Christmas holidays and during Ramadan is considerably lower. The pattern of variation was similar between target, buffer, and control practices. Although this was an opportunistic rather than randomised trial, analysis of variance looked at the impact of time on target and control areas, so demographic and social differences between these practices were taken into account. Although Figure 3 suggests that the AdWords campaign may have had some effect on rates of smears in September and October, analysis of variance on the means in Table 1 showed no significant difference in September and only marginal statistical difference for October between the control and target groups. As Table 2 shows this may be because the standard deviations are quite high.

**Table 2:** Mean rates of smear test per 1000 eligible women in 10 target practice, 90 control practices (more than 2.5 miles away), and 54 control practices (more than 3.5 miles away)

**Area**	**Practices**	**Mean eligible Population per practice**	**Mean (SD) tests per month per thousand eligible women**						
			**May11-Jul11**	**Aug11**	**Sep11**	**Oct11**	**Nov11**	**Dec11**	**Jan12**
Target	10	2088	24.51	18.98	28.66	28.67	22.77	20.43	24.90
			*(4.40)*	*(9.30)*	*(6.07)*	*(14.89)*	*(8.14)*	*(4.06)*	*(10.25)*
Control (≥2.5miles)	90	1466	24.87	20.84	25.90	22.61	26.32	18.54	23.22
			*(6.22)*	*(8.12)*	*(11.05)*	*(7.43)*	*(8.78)*	*(7.35)*	*(7.98)*
Control (≥3.5miles)	54	1455	24.41	21.09	25.67	22.76	26.84	17.90	24.37
			*(5.69)*	*(7.57)*	*(9.27)*	*(7.43)*	*(8.67)*	*(7.26)*	*(8.51)*

If we assumed that the differences in smear rates between target and control group (5.27 per 1000) in September-November 2011 (Table 2) were ‘real’ we might estimate that the intervention resulted in 110 (5.27 * 20.88) extra women being screened. There was no change in coverage levels over May 2011 to January 2012 for intervention, buffer, or control practices. Mean coverage over this nine month period was 69.9% in the target practices, 73.5% in the buffer practices, and 72.0% in control practices. Different sized buffer zones did not impact the statistical significance of the difference between target and control. However, distance from epicentre was a significant predictor in regression analysis suggesting that a design with greater separation between target and control areas may have shown a significant difference in screening rates.

## Discussion

Mass media stories, whether real life or fictional, may raise health awareness among those who are hard to reach by other means. The increase in coverage as a result of the ‘Jade Goody effect’ is evidence of this. Previous fictional events such as in Coronation Street have also helped increase coverage so, as argued by Metcalfe et al., when these occur we need to try to amplify their effect as much as possible [9]. This AdWords campaign aimed to capitalise on public interest generated by an Eastenders’ cervical cancer story. However, although Eastenders remains one of the most popular TV programmes with typical audiences of 8 million [21], this story had little effect on interest in cervical cancer or screening, compared to the large Jade Goody impact. Most Google searches resulting in clicks on our adverts were related to cervical cancer with no mention of Eastenders. So it may be that women clicking on our advert were not the very hard to reach.

Nevertheless, the AdWords campaign may have had some effect. Given the small scale exploratory nature this was not statistically significant but in a larger study, and estimating an effect size of *d* = 0.45 based on the current data, the difference may reach statistical significance. If we make the assumption that the intervention resulted in 110 more women screened for a total cost of £1320 the additional cost per woman screened is £12. So if these rough estimates ‘scaled up’, and if a larger study showed a statistically significant difference resulting from AdWords, the intervention may be cost effective. We cannot estimate from this study what impact an AdWords campaign for cervical screening may have in the absence of media interest but further study may be worthwhile.

We do not know how many people in the East End of London watch Eastenders. It may be that, given the ethnic mix, Eastenders is not that popular, and the initial idea of trying to link a programme with its fictional setting was misguided, but there are no publicly available BBC viewing figures disaggregated to small localities such as the East End to explore this.

We cannot tell if the ‘extra people’ who attended for screening were directly linked to online adverts. Data are not routinely collected from women attending for screening that could be used to indicate whether they were prompted to book an appointment as a result of an external event, or simply as a result of a routine call. Even if women attended as a result of routine calls, this may have been reinforced by online adverts.

Although Metcalfe [9] showed the impact of the Jade Goody case on rates nationally, anecdotally local views in Tower Hamlets were equivocal. On the one hand, some argued that any increase in the number of smear tests at the time of our study might still be due to the Jade Goody effect on the next round of screening. However, although coverage started to increase in the fourth quarter of 2008 it peaked in the second quarter of 2009. The cohort screened at that time would be due for re-screening in the autumn of 2011 through to the summer of 2012 so may have started to have some effect in our study period. On the other hand, a report produced locally by NHS City and Hackney at the time of the ‘Jade Goody’ [7] incident suggested that as the number of women ‘never screened’ did not seem to decrease, the ‘Jade Goody effect’ was just to bring forward the screening date of women who would otherwise have attended.

One of the problems in trying to impact on cervical screening coverage rates in Tower Hamlets is thought to be ‘inflated denominators’ where women who have moved away have not been removed from practice registers. Keeping practice registers up to date is difficult in boroughs such as Tower Hamlets where a quarter of residents move each year [22]. Such problems have been reported by others [23]. On the other hand assessing impact on cervical smears taken is also complicated by the marked seasonal variation. Studies of impact need to examine data over at least one year to control for this variation.

The practices in this study had a mix of ethnicities and these mixes were not the same between target and control practices (Additional file 1). For example, cervical screening rates seem lower amongst Muslim women. In Tower Hamlets PCT 25% of women aged 25–64 were Bangladeshi compared to just under 20% Bangladeshi and Pakistani in Newham. However, our analysis compared change over time and did not directly compare screening rates between areas. The practice populations did not change from the ‘before’ to the ‘during’ period, so any increase in screening for practices in the intervention but not the control could not be explained by differences in the populations.

We think that although this small opportunistic study showed no statistically significant increase in the number of smears or in the coverage, the use of online advertising is worth further study. With a larger study, if there was a reproducible effect, it might be cost effective. Our pilot study of raising awareness of online cognitive behavioural therapy (CBT) for depression [18,24], suggests that AdWords can be effective but the effect is small. So studies need to be correctly sized to have a measurable effect. For the practical reasons that this small evaluation of the use of AdWords in one PCT, using routine data, had to be set up very quickly to take advantage of the Eastenders’ storyline, the control areas were initially selected only on convenience of being within the Tower Hamlets PCT and so under the remit of the authors. This meant that the control areas were only a short distance from the target area. Although our CBT studies have shown that it is possible to run a cluster randomised controlled trial (RCT) without too much contamination, there is still substantial leakage to neighbouring areas [18].

A larger study of online advertising for cervical screening would be worthwhile but would need to include many more geographically dispersed areas. Rather than service evaluation, even though still based on anonymous routinely available statistics, it may require ethical approval. It would require more time to set up, and so it is unlikely that this could be made to coincide with a media event. So a possible further study might be to establish and run a cluster RCT across the UK in areas with currently low coverage, and run it for sufficient time that it might coincide with some media event. For such a study what size sample would be needed? If we assume the effect size of online advertising to be around *d* = 0.40-0.50, based on the current data, we would need approximately 42 target practices and 208 controls to have 90% power of finding a difference. Given that the study design, for practical reasons would likely have to be modified to a cluster RCT, a more practical design would be to use a design we developed elsewhere [18,24]. Such studies are relatively inexpensive.

## Conclusion

This opportunistic study has shown that AdWords run at the time of a media story on cervical cancer may have had some effect and that effect, though small, would be worthwhile. A larger, more appropriately powered cluster RCT study of location targeted online advertising, running for a longer period, in expectation (rather than response to) media stories, would be worthwhile.

## Competing interests

The authors declare that they have no competing interests.

## Authors’ contributions

RJ and MSL had the idea for the project; RJ set up the intervention, analysed the data, wrote the paper; DZ worked with RJ to analyse the data and edited the paper; MSL provided contextual information, obtained routine smear test data, and edited the paper; JS and ETM facilitated the study and edited the paper. All authors read and approved the final manuscript.

## Supplementary Material

Additional file 1: Table S1 Number of practices by PCT in the target, buffer, and control groups (>2.5 miles). **Figure S1.** Ethnicity of women aged 25-64 for the three PCTs. (CH = City and Hackney; N = Newham; TH = Tower Hamlets. **Figure S2. **(Top) deprivation quintiles for three PCTs and (bottom) target for Google AdWords (green circle) and 2.5 mile ‘buffer’ zone’ (red). **Table S2. **Thirteen variations of advert, showing number of presentations, clicks, and click through rate between 3rd August and 22nd October 2011. Click here for file
