# Supplementary material for: Using online adverts to increase the uptake of cervical screening amongst “real Eastenders”: an opportunistic controlled trial
Source: BMC Res Notes. 2013 Mar 26;6:117. doi: 10.1186/1756-0500-6-117 (PMC3621164; doi:10.1186/1756-0500-6-117)
Supplement: Additional file 1: Table S1 — Number of practices by PCT in the target, buffer, and control groups (>2.5 miles). Figure S1. Ethnicity of women aged 25-64 for the three PCTs. (CH = City and Hackney; N = Newham; TH = Tower Hamlets. Figure S2. (Top) deprivation quintiles for three PCTs and (bottom) target for Google AdWords (green circle) and 2.5 mile ‘buffer’ zone’ (red). Table S2. Thirteen variations of advert, showing number of presentations, clicks, and click through rate between 3rd August and 22nd October 2011. [file 1756-0500-6-117-S1.docx]

**Using online adverts to increase the uptake of cervical screening amongst “real Eastenders”: an opportunistic controlled trial**

**Appendix: Additional Tables and Figures**

| Primary Care Trust (PCT) | | GROUP | | | Total |
| --- | --- | --- | --- | --- | --- |
|  |  | Buffer | Target | Control |  |
|  | Tower Hamlets | 19 | 10 | 7 | 36 |
|  | City and Hackney | 26 | 0 | 20 | 46 |
|  | Newham | 0 | 0 | 64 | 64 |
| Total | | 45 | 10 | 91 | 146 |

**Table 1. Number of practices by PCT in the target, buffer, and control groups (>2.5 miles).**

**
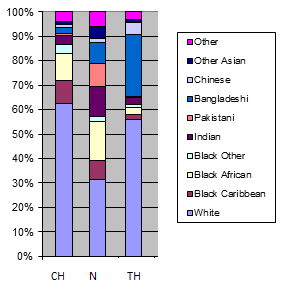
**

**Figure 1. Ethnicity of women aged 25-64 for the three PCTs. (CH = City and Hackney; N=Newham; TH=Tower Hamlets. (**SOURCE: website from ELVIS (East London Common Information Service ) available at <http://nww.elcha.nhs.uk:8080/elvis/login/login.cfm> via NHS net, it is free to use by all NHS staff. For further information, please contact Geoff Mole, senior information analyst, East London Common Information Service on 020 7655 6728 or e-mail [geoff.mole@elcis.nhs.uk](mailto:geoff.mole@elcis.nhs.uk).


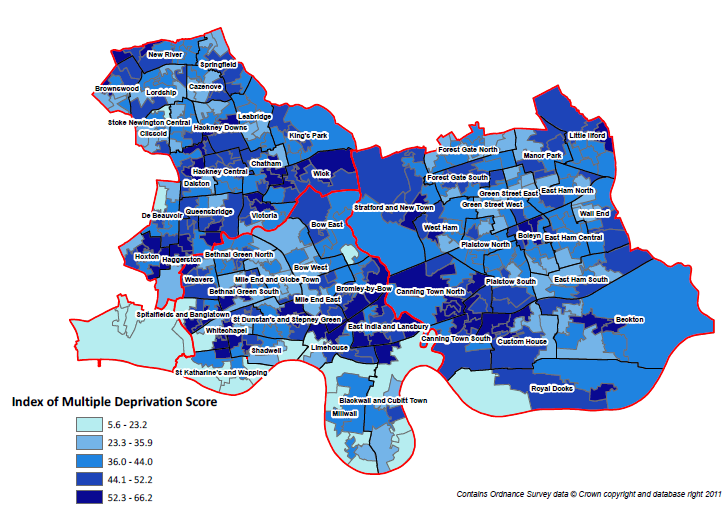


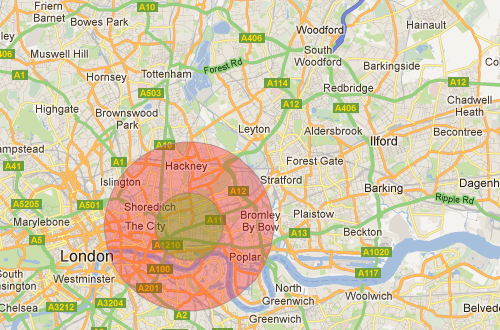


Figure 2. (Top) deprivation quintiles for three PCTs and (bottom) target for Google AdWords (green circle) and 2.5 mile ‘buffer’ zone’ (red).

.

| Advert | Presentations | Clicks | Click through rate |
| --- | --- | --- | --- |
| 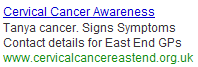 | 5100 | 343 | 6.7% |
| 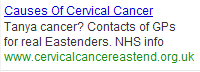 | 1151 | 82 | 7.1% |
| 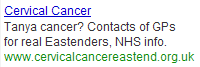 | 3463 | 78 | 2.3% |
| 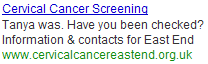 | 1524 | 71 | 4.7% |
| 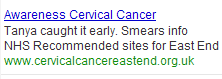 | 786 | 44 | 5.6% |
| 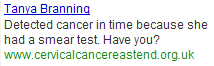 | 1172 | 30 | 2.6% |
| 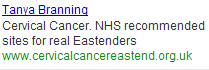 | 659 | 20 | 3.0% |
| 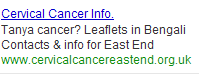 | 976 | 17 | 1.7% |
| 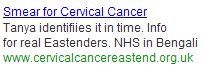 | 245 | 12 | 4.9% |
| 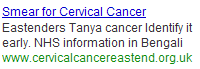 | 186 | 9 | 4.8% |
| 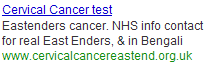 | 404 | 9 | 2.2% |
| 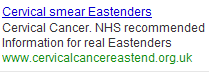 | 156 | 1 | 0.6% |
| 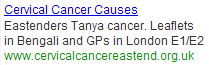 | 72 | 0 | 0 |

**Table 2. Thirteen variations of advert, showing number of presentations, clicks, and click through rate between 3^rd^ August and 22^nd^ October 2011.**
